# Optimization of Pectin Enzymatic Extraction from *Malus domestica* ‘Fălticeni’ Apple Pomace with Celluclast 1.5L

**DOI:** 10.3390/molecules24112158

**Published:** 2019-06-07

**Authors:** Florina Dranca, Mircea Oroian

**Affiliations:** Faculty of Food Engineering, Stefan cel Mare University of Suceava, Suceava 720229, Romania; florina.dranca@usm.ro

**Keywords:** pectin extraction, apple, *Malus domestica* ‘Fălticeni’, Celluclast, optimization

## Abstract

Pectin was extracted from apple (*Malus domestica* ‘Fălticeni’) pomace with Celluclast 1.5L, at doses of 20, 40, and 60 µL/g of material. The temperature and time of extraction were varied on three levels—temperature—40, 50, and 60 °C; time—12, 18, and 24 h. For each experiment, the extraction yield (R^2^ = 0.8905), the galacturonic acid content (R^2^ = 0.9866), and the degree of esterification (R^2^ = 0.9520) of pectin was determined. Response surface methodology (RSM) was implemented via a Box–Behnken design, to optimize pectin extraction. In the optimum extraction conditions (temperature of 48.3 °C, extraction time of 18 h 14 min, and enzyme dose of 42.5 µL/g of pomace), the design predicted a 6.76% yield with a galacturonic acid content of 97.46 g/100 g of pectin and a degree of esterification of 96.02%. FT-IR analysis of the pectin sample obtained in these conditions showed a chemical structure similar to that of commercial apple and citrus pectin.

## 1. Introduction

Pectins are complex polysaccharides that are widely distributed in the primary cell walls and the middle lamella of higher plants, having important functions in their development, growth, and maturation [1,2]. The three main domains identified and characterized in the structure of pectin are homogalacturonan (HG), rhamnogalacturonan type I (RG-I), and rhamnogalacturonan type II (RG-II). HG, which is the most abundant pectic polysaccharide that makes up approximately 50%–90% of the total pectin, is a linear polymer of *d*-galacturonic acid units that are α(1→4) linked, partial methyl-esterified at the C-6 carboxyl, and depending on the plant source, *O*-acetylated at the O-2 or O-3 positions [2,3]. The second most predominant pectic polysaccharide is RG-I (20%–35% of the total pectin), which is formed of *d*-galacturonic acid units interspersed by α(1→2) linked, *L*-rhamnose units, which can be substituted with side chains of arabinans, galactans, and arabinogalactans [4]. RG-II is a branched polygalacturonate polymer that contains complex side chain sugar moieties [5]. The overall structure of pectin and its chemical composition govern its applications [6].

The main commercial sources for pectin extraction are citrus peel and apple pomace, however, the increasing industrial demand and the growing interest in valorizing side streams, to obtain pectins with diverse functional properties, have led to an extensive evaluation of alternative pectin sources [6]. Some of the new pectin sources studied in the last years were pumpkin waste, watermelon rinds [7], banana peel [8], pistachio green hull [9], cocoa pod husks [10], and sisal waste [11].

The conventional extraction methods applied to obtain pectin involve the use of water, together with chemicals such as complexing compounds, acids, or alkali. On an industrial scale, pectin is produced from apple pomace or citrus fruit peel through extraction in warm-diluted solutions of mineral acids (hydrochloric, nitric, or sulfuric acid) at a pH ranging from 1 to 3, at temperatures of 60–100 °C, and 1–12 h extraction time, depending on the source material and the desired physicochemical properties of the extracted pectin [4]. In an attempt to develop green procedures of processing fruit and vegetable by-products and waste for the extraction of pectin, milder extraction agents (e.g., citric acids and enzymes) and non-conventional extraction techniques (such as microwave- and ultrasound-assisted extraction) have been investigated. The use of enzymes for pectin extraction might bring two major advantages related to selectivity and efficiency, especially in terms of the extraction yield. Two main approaches are distinguished in enzyme-assisted extraction—(a) the use of enzymes that degrade and isolate specific pectin fragments and (b) the use of enzymes capable of deconstructing the plant cell wall and isolating the whole pectin molecule [12,13].

In this study, we took the second approach to enzyme-assisted extraction by investigating the use of enzymes that degrade the plant cell wall component to extract pectin. For this purpose, we analyzed the efficiency of the commercially available multicatalytic enzyme preparation Celluclast 1.5L in apple pectin extraction. The plant material used as a source of pectin was apple pomace obtained by locally processing apples from the autumn variety *Malus domestica* ‘Fălticeni’. *Malus domestica* ‘Fălticeni’ apple, developed in the geographical area of Fălticeni, Suceava (Romania), is a late season variety suitable for industrial processing, which generates the premises for the capitalization of the by-product resulted from the process. The extraction of pectin was conducted on a laboratory scale, and the influence of the enzyme dose, as well as that of the extraction temperature and time, was studied.

## 2. Results and Discussion

Pectin samples were extracted and purified by means of the procedure described in Section 3.2 and Section 3.3. The applied enzyme-assisted extraction with Celluclast 1.5L is a green procedure that eliminates the major disadvantages of the conventional acid extraction of pectin—the high temperatures and the low pH of the extracting solution. The lower temperatures of enzymatic extraction lead to a reduction of energy consumption and, thus, lower operational costs. By using citric acid instead of strong mineral acids (such as hydrochloric or sulfuric acid) to bring up the pH to a value of 4.5 (which is required for an optimum activity of the enzymatic preparation) the high costs incurred by the treatment of the strong acidic waste that resulted from the extraction process are avoided. Apart from these improvements, the green extraction method should provide a high-quality extract. The influence of the conditions of enzyme-assisted extraction with Celluclast 1.5L, on the pectin yield and its composition, are discussed below.

### 2.1. Model Fitting and Statistical Analysis

With the purpose of studying and optimizing the extraction of pectin from *Malus domestica* ‘Fălticeni’ pomace, a Box–Behnken design was used, which had three independent variables, each with three levels and three replications at the center point. For the extraction of pectin from this vegetal source, a complex process was applied that allowed the variation in the levels of the three process parameters—temperature (T, °C), time (t, h), and enzyme dose (Ed, µL/g). Table 1 presents the predicted response and the experimental values obtained for the yield (Y), the galacturonic acid content (GalA), and the degree of esterification (DE), as a result of varying the conditions of pectin extraction.

As the quadratic model showed higher values of coefficient of determination (R^2^), adjusted R^2^, and also showed low *p*-values (especially for the galacturonic acid content and the degree of esterification, when compared to the linear and 2FI models), this model was selected to study the effect of the independent variables on the pectin yield and the physicochemical properties. According to the results of the analysis of variance (Table 2), the coefficients of determination obtained for the pectin yield, the galacturonic acid content, and the degree of esterification (0.8905, 0.9866, and 0.9520, respectively) indicate that the quadratic model could explain and predict most of the variation of these responses.

The second-order polynomial equations describing the effect of the independent variables considered on the responses—pectin yield (Equation (1)), galacturonic acid content (Equation (2)), and degree of esterification (Equation (3)) were as follows:

Y (%) = 6.579 − 0.472 × *T* + 0.667 × *t* + 0.862 × *Ed* − 0.163 × *T* × *t* − 0.148 × *T* × *Ed* + 0.802 × *t* × *Ed* − 0.820 × *T*^2^ − 0.458 × *t*^2^ − 0.458 × *Ed*^2^ (1)

GalA (g/100 g) = 99.451 + 3.901 × *T* − 8.279 × *t* − 5.770 × *Ed* + 3.967 × *T* × *t* − 3.735 × *T* × *Ed* − 5.612 × *t* × *Ed* − 8.660 × *T*^2^ − 4.686 × *t*^2^ − 4.992 × *Ed*^2^ (2)

DE (%) = 95.770 − 1.500 × *T* + 0.653 × *t* + 0.416 × *Ed* − 0.780 × *T* × *t* + 1.230 × *T* × *Ed* − 1.047 × *t* × *Ed* − 1.573 × *T*^2^ − 0.261 × *t*^2^ − 0.076 × *Ed*^2^ (3)

### 2.2. Effect of the Extraction Conditions on Pectin Recovery and the Physicochemical Properties

#### 2.2.1. Influence of the Extraction Parameters on the Yield

**Figure 1:**
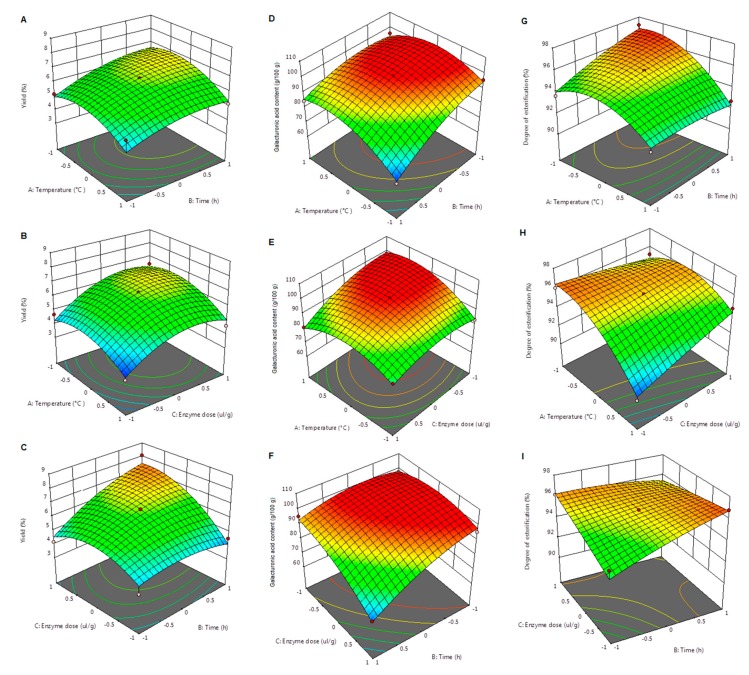
Response surface plots showing the effect of extraction parameters on the pectin yield (**A**–**C**), the galacturonic acid content (**D**–**F**), and the degree of esterification (**G**–**I**).

As seen from results presented in Table 1, the measured pectin yield varied between the lowest value of 3.40% and the maximum value of 8.08%. The conditions that led to the highest pectin recovery from the plant material were as follows—enzyme dose of 60 µL/g of apple pomace, temperature of 50 °C, and 24 h extraction time. Table 2 shows the influence of each parameter, as well as the interactions between the process parameters and their statistical significance. The evolution of the extraction yield in the function of temperature, time, and enzyme dose is presented in Figure 1A–C.

The extraction temperature is an important parameter of enzyme-assisted extraction that was previously shown to have a strong influence on the pectin yield. In the works of Pasandide et al. [14] and Geng et al. [15], the increase of temperature from 70 to 90 °C and from 80 to 100 °C, respectively, led to a maximum extraction yield. The influence of this parameter in the case of our study was completely different from the results of these two studies. As shown in Figure 1A,B, the increase of temperature did not cause a positive effect on pectin yield. Moreover, according to the data in Table 3, the extraction temperature did not significantly affect the extraction yield (*p* > 0.05). The best results were obtained when the temperature was 50 °C—an observation that was in accordance with the optimal value for the temperature of enzymatic pectin extraction with Celluclast 1.5L, as found by Wikiera et al. [16].

In this study, the influence of the extraction time on pectin yield was less pronounced; however, this working parameter was still a statistically significant factor (*p* < 0.05). When the temperature was fixed to 50 °C (Figure 1C), pectin yield showed an increase with the increase of both the extraction time and the enzyme dose. The interaction between these two parameters also proved to be statistically significant (*p* < 0.05). An increase of the pectin yield with extraction time was also reported by Pasandide et al. [14].

Unlike time and temperature, which had little or no influence on the yield, the extraction of pectin from apple pomace was significantly influenced by the enzyme dose (*p* < 0.01). As the 3D graph in Figure 1C shows, together with the increase of extraction time, the increase in enzyme dose led to a substantial increase of pectin yield from 3.80% (20 µL/g of apple pomace; 12 h extraction time) to 8.08% (60 µL/g of apple pomace; 24 h extraction time). Although the increase of pectin yield was substantial, the maximum recovery reached in this study was way below the minimum value of 15.3% [17] and the level of 18.95% [16] reported for the use of Celluclast 1.5L for the apple pectin extraction. Furthermore, the maximum pectin yield was more than three times lower than the maximum yield of 21.24% that we obtained for the extraction of pectin from *Malus domestica* ‘Fălticeni’ using citric acid [18]. Although the maximum yield was obtained with the highest enzyme dose, suggesting that a further increase of this parameter might result in a higher pectin recovery from the plant material, some published studies have shown that Celluclast 1.5L should be added in small quantities because it could cleavage some of the linkages between the galacturonic acid residues [19,20]. Thus, this difference between the yield of acid extraction and that of the enzyme-assisted extraction can be attributed to some degradation in the carbohydrate structure that takes place simultaneous to the action of the multicatalytic enzymatic preparation upon the cell wall components surrounding the pectin molecule.

#### 2.2.2. Effect of the Extraction Conditions on the Content of Galacturonic Acid of Pectin

As shown in Table 1, the content of galacturonic acid determined in the pectin extracted from *Malus domestica* ‘Fălticeni’ pomace varied between 68.21 g/100 g pectin (temperature, 40 °C; time, 24 h; and enzyme dose, 40 µL/g pomace) and 99.45 g/100 g pectin (temperature, 50 °C; time, 18 h; and enzyme dose, 40 µL/g pomace). Table 2 presents the influence of the extraction conditions on this chemical parameter, as well as the interactions between parameters and their statistical significance. The changes in the content of galacturonic acid determined by the extraction conditions are visualized in the 3D graphics of Figure 1D–F.

The extraction temperature was the first parameter that showed a significant influence (*p* < 0.001) on the galacturonic acid content. As shown in Figure 1D,E, the increase of temperature led to a higher galacturonic acid content in the extracted pectin. A similar effect of the extraction temperature on this chemical parameter was also reported by Priyangini et al. [10], when studying the extraction of pectin from cocoa pod husks. Regarding the effect of the interactions between temperature and other extraction parameters on the galacturonic acid content (Table 3), a strong influence (*p* < 0.01) of the interaction of temperature–enzyme dose and temperature–extraction time was noted. This last extraction parameter manifested an opposite effect on the galacturonic acid content, when compared to the influence of temperature. Figure 1D,F show that the increase in extraction time resulted in a decrease of the galacturonic acid content of pectin. The same effect of a prolonged extraction time was also observed by Colodel et al. [4] in a study on the extraction of pectin from ponkan peel. A different conclusion with respect to the effect of this parameter was stated in the study of Vriesmann et al. [3], who found that the extraction of pectin from cacao pod was not significantly influenced by the pH and the extraction time.

Similar to the influence of time, the increase of enzyme dose caused a decrease in the galacturonic acid content of apple pectin (Figure 1E,F). This effect was opposite to that manifested by enzyme dose on the extraction yield. As presented in Table 2, the interaction between the two parameters whose increase led to a decrease of the content of galacturonic acid (namely time and enzyme dose) was statistically significant (*p* < 0.001). The maximum value for the galacturonic acid content of pectin extracted with Celluclast 1.5L was higher (99.45 g/100 g versus 93.90 g/100 g) than that determined for pectin extracted from *Malus domestica* ‘Fălticeni’ using citric acid [18]. A slightly higher galacturonic acid content of the pectin extracted with this enzyme preparation, in comparison to that extracted with sulfuric acid, was also reported by Wikiera et al. [16].

#### 2.2.3. Influence of the Extraction Conditions on the Degree of Esterification of Pectin

The data presented in Table 1 shows that the various levels of the extraction parameters determined variations in the degree of esterification of apple pectin, between a minimum value of 90.55% (temperature, 60 °C; time, 18 h; and enzyme dose, 20 µL/g pomace) and a maximum of 97.21% (temperature, 40 °C; time, 24 h; and enzyme dose, 40 µL/g pomace). Considering the classification of pectin according to the degree of esterification [21], all samples of apple pectin obtained in this study were high-esterified. Although no great variation was recorded for the degree of esterification, the influence of the extraction conditions on this parameter was a subject of interest for the research, particularly from the point of view of comparing the effect of the extraction parameters on the degree of esterification to that manifested on the pectin yield and its galacturonic acid content. The evolution of the degree of esterification in the function of temperature, time, and enzyme dose is presented in Figure 1G–I.

As presented in Table 2, the extraction temperature was the most important parameter that greatly influenced the degree of esterification of the extracted pectin (*p* < 0.001). Figure 1G shows an increase of the degree of esterification when the extraction temperature decreased and the extraction time increased (the enzyme dose was fixed to 40 µL/g pomace). The interaction between these two parameters was statistically significant (*p* < 0.05). A similar influence of temperature on the degree of esterification was reported by Pasandide et al. [14], for *Citrus medica* peel pectin. As mentioned before, the increase in extraction time had a positive influence on the degree of esterification (*p* < 0.05); however, this observation was not corroborated with previous studies that concluded in the finding that harsh extraction conditions such as high temperature and pH, and prolonged extraction, could determine increased de-esterification of the polygalacturonic chain [22].

Regarding the influence of enzyme dose, Table 2 shows that this working parameter did not significantly affect the degree of esterification of the extracted apple pectin (*p* > 0.05). However, the interactions of enzyme dose–temperature and enzyme dose–time were statistically significant (*p* < 0.01). Figure 1H,I indicate that the degree of esterification determined for the pectin samples was higher when lower enzyme doses were used for the extraction. The difference was more pronounced in the case of the variation of the degree of esterification determined by the temperature and enzyme dose interaction (time was fixed to 18 h).

### 2.3. Optimization of Extraction Parameters

In this study, the extraction conditions (temperature, time, and enzyme dose) were optimized, such that the pectin yield and its galacturonic acid content and degree of esterification was maximized. By using the response surface methodology (RSM) for optimization, the software used in this study predicted that a temperature of 48.3 °C, extraction time of 18 h 14 min, and enzyme dose of 42.5 µL/g pomace were the optimal conditions to obtain a 6.76% pectin yield, with a content of galacturonic acid of 97.46 g/100 g pectin and a degree of esterification of 96.02%.

### 2.4. Structure Analysis of Pectin by FT-IR

**Figure 2:**
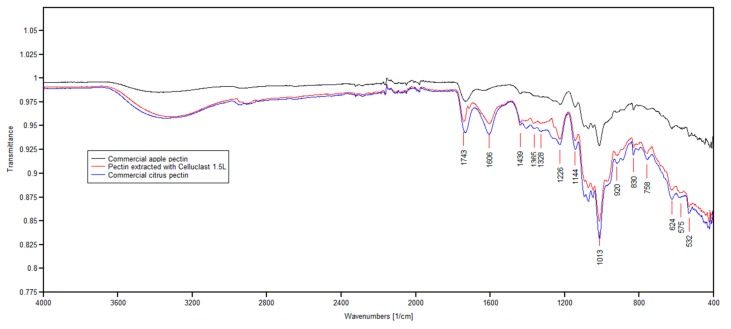
Spectra of the pectin extracted from apple (*Malus domestica* ‘Fălticeni’) pomace with Celluclast 1.5L under optimal conditions and commercial apple and citrus pectin.

Figure 2 shows the infrared spectra of pectin extracted from the *Malus domestica* ‘Fălticeni’ apple, using the multicatalytic enzyme preparation Celluclast 1.5L under the optimal conditions presented in Section 2.3, together with the spectra obtained for commercial apple and citrus pectin. By comparison with previous studies on the FT-IR spectra of pectin [23,24], for the three samples that were analyzed in this study, no major peaks were found in the region of 3600–3000 cm^−1^. The peak at 1743 cm^−1^ indicated ester carbonyl (C=O) stretching, while 1606 cm^−1^ was attributed to carboxylate ion stretching [25]; the ratio between these peaks could be used for the quantification of the degree of esterification. Based on the absorption spectra, the apple pectin sample extracted with Celluclast 1.5L was high-esterified and was found to be more similar to the commercial citrus pectin than commercial apple pectin, in terms of the degree of esterification.

Besides the strong asymmetrical stretching at 1606 cm^−1^, the carbohydrate groups showed another weak symmetric stretching band around 1439 cm^−1^. This was followed by intense absorption patterns between 1300 and 800 cm^−1^. These are collectively referred to as the ‘finger print’ region for carbohydrates because it is unique to a compound and, therefore, allows the identification of major chemical groups in polysaccharides [26]. In this region, the bands between 1120–990 cm^−1^ were identified as the range for the spectral identification of galacturonic acid in pectin molecules [27]. The high intensity peak at 1013 cm^−1^ indicated that the apple pectin sample extracted with Celluclast 1.5L contains pyranose, and the peaks at 920 cm^−1^ and 830 cm^−1^ referred to the absorption of *d*-glucopyranosyl and α-*d*-mannopyranose, respectively; similar findings were reported in previous studies by Wang et al. [28] and Zhang et al. [29].

### 2.5. Thermal Properties

**Figure 3:**
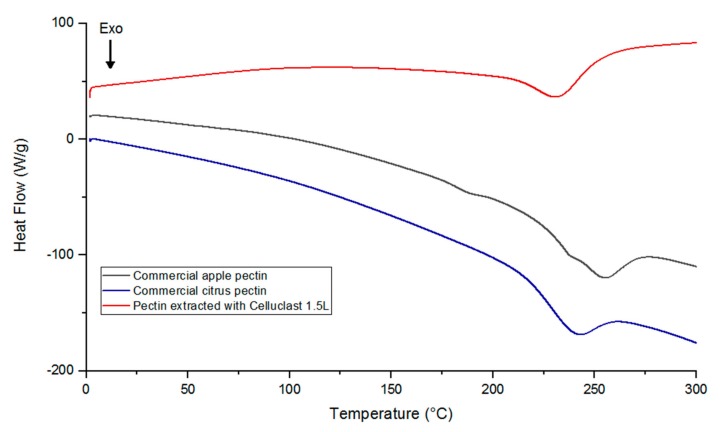
Differential scanning calorimetry (DSC) thermograms of the pectin extracted with Celluclast 1.5L under optimal conditions, and that for commercial apple and citrus pectin.

With the purpose of investigating the thermal behavior of pectin extracted from *Malus domestica* ‘Fălticeni’ apple pomace with the multicatalytic enzyme preparation Celluclast 1.5L (under the optimal conditions) and to compare it with that of commercial apple and citrus pectin, differential scanning calorimetry (DSC) analysis was conducted. As shown in the thermograms presented in Figure 3, for the samples of commercial pectin, no endothermic peaks (melting temperature) were observed, while exothermic peaks (degradation temperature) were recorded at 243 °C for citrus pectin and at 255 °C for apple pectin. According to previous studies, endothermic peaks indicated the presence of water in the analyzed sample, hydrogen bonding among the galacturonic acid units, and also conformational changes of the galacturonan ring [28,30]. In the case of this study, the lack of an endothermic peak could indicate that no breakage of hydrogen bonds occurred in the structure of these pectin samples. Conversely, the exothermic peaks of commercial apple and citrus pectin were close to those reported in the works of Wang et al. [23,31], indicating a similar profile of chemical constituents.

Regarding the thermal behavior of the pectin sample extracted from *Malus domestica* ‘Fălticeni’ apple pomace, Figure 3 shows a very small endothermic peak at 120 °C and an exothermic peak at 230 °C, which were in accordance with the melting and degradation temperatures recorded by Wang et al. [23]. When compared to the commercial apple pectin sample, pectin extracted with Celluclast 1.5L had a lower degradation temperature that might be explained by some differences in its chemical structure, which were also observed in the FT-IR analysis. However, considering the intensity of the flow of heat, thermal analysis showed that the sample obtained from apple pomace by enzymatic extraction possessed a higher thermal stability than commercial apple and citrus pectins, which showed more significant changes during heating. From the point of view of the use of pectin in food industry, this might suggest good behavior during thermal processing.

## 3. Material and Methods

### 3.1. Materials

Apple pomace was obtained in a small scale plant by processing apples (*Malus domestica* ‘Fălticeni’) from the 2016 harvest, in the Fălticeni area of Suceava, Romania. The pomace was dried in an oven with air circulation at 60 °C, until constant weight, and then powdered in a food processor. The resulting powder was passed through an analytical sieve shaker Retsch AS 200 (Retsch GmbH, Haan, Germany) and the apple pomace with particle sizes of 125–200 µm was used for the pectin extraction.

Celluclast 1.5L, citric acid, ethyl alcohol, sulfamic acid, potassium hydroxide, sulfuric acid, sodium tetraborate, sodium hydroxide, *d*-galacturonic acid, *m*-hydroxydiphenyl, and commercial apple and citrus pectin were purchased from Merck KGaA (Darmstadt, Germany).

### 3.2. Enzyme-Assisted Extraction of Apple Pectin

**Figure 4:**
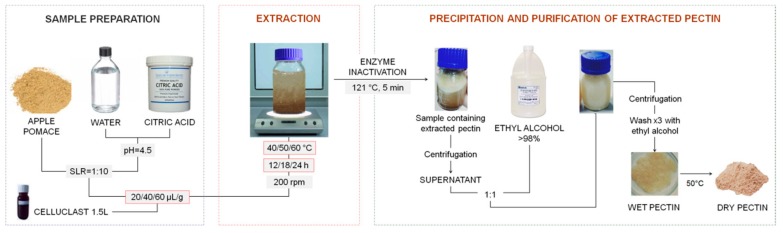
Procedure applied for the enzyme-assisted extraction of pectin from apple (*Malus domestica* ‘Fălticeni’) pomace with Celluclast 1.5L; process parameters are marked by red border.

The procedure followed to obtain the pectin from apple (*Malus domestica* ‘Fălticeni’) pomace with Celluclast 1.5L is presented in Figure 4. In the first step, the water used for the extraction was brought to a pH of 4.5 with citric acid. Then, the extraction mixture was prepared by adding 4.5 of apple pomace to 45 mL of water, keeping a solid-to-liquid ratio of 1:10. Enzyme doses of 20, 40, and 60 µL/g of apple pomace, respectively, were added to the water–pomace mixture, according to the Box–Behnken design presented in Table 3. The extraction process was conducted at different temperatures (40, 50, and 60 °C) and times (12, 18, and 24 h) with constant shaking (200 rpm).

### 3.3. Pectin Precipitation and Purification

After extraction, the enzyme was inactivated at 121 °C for 5 min and the samples were cooled to the room temperature, prior to precipitation and purification. First, a centrifugation at 4000 rpm for 40 min was performed to separate pectin from the remaining solid material. The supernatant was filtered through a clean cheesecloth folded 6 times, transferred in a laboratory glass bottle, and mixed with cold (4 °C) ethyl alcohol (>96%, *v*/*v*), with the volume added being calculated to reach a final concentration of alcohol of 70%. The mixture was kept at 4–6 °C for 12 h to complete the precipitation. Then, the precipitated pectin was separated from the mixture, by centrifugation, under the same conditions as mentioned above. The wet pectin was washed three times, with 96% ethyl alcohol, and finally dried in a hot air oven, at 50 °C, to a constant weight.

The extraction efficiency was calculated using Equation (4):

(4) $$Pectin\;yield\text{​ }(\%)=\frac{m_{0}}{m}\times 100$$

where, *m*_0_—weight of dried pectin (g) and *m*—weight of dried apple pomace powder (g).

### 3.4. Pectin Characterization

#### 3.4.1. Galacturonic Acid Assay

The content of galacturonic acid was measured using the sulfamate/*m*-hydroxydiphenyl method developed by Filisetti-Cozzi et al. [32]. The preparation of the samples was made according to Miceli-Garcia [33], as follows—20 mg of pectin were mixed with 50 mL of distilled water (at 40 °C) under constant shaking (400 rpm), until the sample was completely dispersed, then the volume was adjusted to 100 mL with warm distilled water. From the pectin solution, aliquots of 400 μL were placed in glass tubes, to which 40 μL of 4 M sulfamic acid solution followed by 2.4 mL of sulfuric acid containing 75 mM of sodium tetraborate were added, while vigorously vortexing the content of the tubes for at least 5 s, in between the additions. The samples were boiled for 20 min in a water bath, and then cooled for 10 min in an ice bath. A total of 80 μL of *m*-hydroxydiphenyl solution in 0.5% (*w*/*v*) sodium hydroxide were added to each test tube and the content was vortex mixed, and after 10–30 min, the absorbance was read at 525 nm, against the reagent control (prepared following the same methodology as described above, but replacing the *m*-hydroxydiphenyl solution with 0.5% (*w*/*v*) sodium hydroxide) with a UV-Vis-NIR spectrophotometer (Shimadzu Corporation, Kyoto, Japan). A standard curve of *d*-galacturonic acid was made for each batch of samples that was analyzed.

#### 3.4.2. Determination of the Degree of Esterification of Pectin

For the determination of the degree of esterification of the extracted pectin, the titrimetric method previously described by Franchi et al. [34] was used. In brief, 50 mg of pectin were dissolved in 10 mL of boiled distilled water and the resulting solution was titrated with 0.1 N NaOH, using phenolphthalein as an indicator; the volume of used sodium hydroxide solution was recorded as *V*_1_. Subsequently, 20 mL of 0.5 M NaOH were added and the solution was allowed to stand for 30 min. A total of 20 mL of 0.5 M HCl were subsequently added to neutralize the solution under vigorous stirring, and a final titration with 0.1 N NaOH was made, recording the sodium hydroxide volume as *V*_2_. The degree of esterification was calculated using Equation (5):

(5) $$Degree\;of\;esterification\;(\%)=\frac{V_{2}}{V_{1}+V_{2}}\times 100$$

#### 3.4.3. Fourier Transform Infrared (FT-IR) Spectroscopy Analysis

The pectin sample extracted with Celluclast 1.5L in optimal conditions and the commercial apple and citrus pectin samples were subjected to FT-IR spectroscopy, using a Fourier-Transform Infrared Spectrophotometer (Spectrum Two; Perkin Elmer, Waltham, Massachusetts, MA, USA). The characteristic spectra of the pectin samples were recorded in the range 4000–400 cm^−1^ at a resolution of 4 cm^−1^ [28]. Spekwin32–optical spectroscopy software (Version 1.72.2, Dr. Friedrich Menges Softwareentwicklung, Oberstdorf, Germany) was used to display the spectra.

#### 3.4.4. Thermal Analysis

Differential scanning calorimetry (DSC 8500; Perkin Elmer) was used in this study to investigate the thermal properties of the pectin sample extracted with Celluclast 1.5L (under optimal conditions) and compare them with those of the commercial apple and citrus pectin samples. From each sample, 5 mg of dried powder were added to the aluminum pans that were immediately sealed and placed in the DSC instrument, together with an empty aluminum pan that was used as a reference. The change in heat flow was analyzed from 0 °C to 300 °C (heating rate of 10 °C/min) in dynamic inert nitrogen atmosphere, at a flow rate of 20 mL/min.

### 3.5. Experimental Design

The effect of the independent variables (temperature (T), time (t), and enzyme dose (Ed)) on the extraction yield, galacturonic acid content, and degree of esterification was monitored using a Box–Behnken response surface experimental design. The coded levels of the design variables are shown in Table 3. All computation and graphics were made using Design Expert v11 (trial version, Stat-Ease, Minneapolis, Minnesota, MN, USA).

## 4. Conclusions

The pomace obtained by processing apples from the autumn variety *Malus domestica* ‘Fălticeni’, in the geographical area of Fălticeni, Suceava, Romania, was found to be a good source material for pectin extraction. For the extraction, the commercially available multicatalytic enzyme preparation, Celluclast 1.5L, was used, and the following process parameters were varied—temperature, time, and enzyme dose. The effect of these parameters was studied by recording the changes in pectin yield and its galacturonic acid content and the degree of esterification. Pectin yield was found to be strongly influenced by enzyme dose, while temperature and extraction time had little or no effect on the extraction. Regarding the effect of process parameters on the galacturonic acid content of pectin, the increase in temperature was found to have a positive influence, but that of time and enzyme dose had a negative impact on this parameter. Although all extracted samples were classified as high-esterified, those that had higher degree of esterification were obtained at a low temperature and a prolonged extraction time. FT-IR spectra showed that the pectin sample extracted under optimal conditions had a chemical structure similar to that of commercial citrus and apple pectin, while DSC analysis revealed a high thermal stability of this sample.

As a final remark, it is important to address the prospects for the industrial-scale application of enzyme-assisted extraction of pectin from *Malus domestica* ‘Fălticeni’ apple pomace. This application successfully meets the important conditions for industrialization, which are the production of high-quality pectin and the use of a sustainable extraction process that involves the addition of a reduced quantity of cheap multicatalytic preparation Celluclast 1.5L, and eliminates the requirement of a low pH and high extraction temperatures. In this way, the possibility of corrosion, as well as rapid wearing out of the equipment would no longer present as a problem. From this point of view, pectin production with Celluclast 1.5L fits the trend of environment-friendly extraction technologies.

## Figures and Tables

**Table 1:** Box–Behnken design with the experimental values and predicted values.

Run.	Independent Variables			Measured Response			Predicted Response		
	Ed	T	t	Y (%)	GalA (g/100 g)	DE (%)	Y (%)	GalA (g/100 g)	DE (%)
1	20	50	24	4.51	95.20	96.78	4.14	92.88	96.72
2	40	40	12	5.13	95.66	93.64	4.94	94.45	94.00
3	40	50	18	6.58	99.45	95.77	6.56	99.44	95.78
4	20	50	12	3.80	97.58	94.08	4.41	98.21	93.32
5	60	50	12	4.16	95.57	96.18	4.53	97.89	96.24
6	40	60	24	5.15	84.49	92.67	5.33	85.69	92.31
7	40	50	18	6.58	99.45	95.77	6.56	99.44	95.78
8	40	50	18	6.58	99.45	95.77	6.56	99.44	95.78
9	20	40	18	4.66	83.35	96.03	4.24	83.93	96.43
10	20	60	18	3.40	98.08	90.55	3.59	99.20	90.97
11	40	60	12	5.12	96.06	92.22	4.32	94.32	92.56
12	60	60	18	4.59	80.77	94.67	5.01	80.19	94.27
13	40	40	24	5.81	68.21	97.21	6.60	69.96	96.87
14	60	50	24	8.08	70.74	94.69	7.47	70.11	95.46
15	60	40	18	6.44	80.98	95.23	6.26	79.86	94.81

**Table 2:** Analysis of variance (ANOVA) for yield, galacturonic acid content, and degree of esterification of the pectin extracted from *Malus domestica* ‘Fălticeni’ pomace.

Source	Sum of Squares	DF	Mean Square	*F*-Value	*p*-Value
(A) Pectin yield, %					
Model	22.71	9	2.52	6.33	0.0119
Temperature	1.79	1	1.79	4.49	0.0719
Time	3.57	1	3.57	8.94	0.0202
Enzyme dose	5.95	1	5.95	14.93	0.0062
Temperature × time	0.10	1	0.10	0.26	0.6211
Temperature × enzyme dose	0.08	1	0.08	0.22	0.6529
Time × enzyme dose	2.58	1	2.58	6.46	0.0386
R^2^			0.8905		
Adjusted R^2^			0.7497		
(B) Galacturonic acid content, g/100 g					
Model	1747.93	9	194.21	57.08	<0.0001
Temperature	121.76	1	121.76	35.78	0.0006
Time	548.36	1	548.36	161.16	<0.0001
Enzyme dose	266.34	1	266.34	78.28	<0.0001
Temperature × time	62.96	1	62.96	18.50	0.0036
Temperature × enzyme dose	55.82	1	55.82	16.40	0.0049
Time × enzyme dose	125.99	1	125.99	37.03	0.0005
R^2^			0.9866		
Adjusted R^2^			0.9693		
(C) Degree of esterification, %					
Model	46.74	9	5.19	15.42	0.0008
Temperature	18.00	1	18.00	53.45	0.0002
Time	3.42	1	3.42	10.15	0.0154
Enzyme dose	1.39	1	1.39	4.12	0.0821
Temperature × time	2.43	1	2.43	7.23	0.0312
Temperature × enzyme dose	6.05	1	6.05	17.97	0.0038
Time × enzyme dose	4.39	1	4.39	13.03	0.0086
R^2^			0.9520		
Adjusted R^2^			0.8903		

**Table 3:** Levels of the independent variables applied in the Box–Behnken design.

Factor	Coded Symbols	Levels		
		−1	0	1
Temperature (°C)	T	40	50	60
Extraction time (h)	t	12	18	24
Enzyme dose (µL/g apple pomace)	Ed	20	40	60
